# Case Report: Transferable IncX4 plasmid carrying *mcr-1* in colistin-resistant *Escherichia coli* from a healthy pet dog in South Korea

**DOI:** 10.3389/fvets.2025.1746399

**Published:** 2026-01-27

**Authors:** Jae Young Oh, Su Min Kwak, Joo Yeol Kim, Woong-Bin Ro, Kwang Jun Lee, Jong-Chan Chae

**Affiliations:** 1 Division of Biotechnology, Jeonbuk National University, Iksan, Republic of Korea; 2 Advanced Institute of Environment and Bioscience, Jeonbuk National University, Iksan, Republic of Korea; 3 Department of Veterinary Emergency and Critical Care Medicine, College of Veterinary Medicine, Chonnam National University, Gwangju, Republic of Korea; 4 Division of Zoonotic and Vector Borne Disease Research, National Institute of Health, Cheongju, Republic of Korea

**Keywords:** colistin resistance, companion dog, *Escherichia coli*, IncX4 plasmid, *mcr-1*

## Abstract

Colistin resistance mediated by the plasmid-borne *mcr-1* gene presents a significant challenge in both human and veterinary medicine. While colistin-resistant bacteria have been reported in food-producing animals and humans, *mcr-1*-harboring strains remain relatively underreported in companion animals, particularly in South Korea. In this study, a colistin-resistant *Escherichia coli* strain Z1324PEC0026 was isolated from a clinically healthy companion dog owned by a veterinary nurse, which exhibited resistance to multiple antimicrobials, including colistin, β-lactams, aminoglycosides, tetracyclines, and phenicols. Its genome harbored two plasmids: a 33.9 kb IncX4 plasmid pEC027-3 carrying *mcr-1* gene with a high conjugation frequency, 4.64 × 10^−2^ per recipient cell, and another plasmid pEC027-2 carrying additional resistance genes such as *bla*_CTX-M-55_, *bla*_OXA-10_, *qnrS1*, *dfrA14*, *aph(3”)-Ia*, *aadA1*, *cmlA1*, *arr-2*, and *tet(A)*. The genetic structure of pEC027-3 exhibited high synteny with global IncX4 plasmids but showed divergence from *mcr-1*-carrying plasmids previously reported in South Korea, suggesting an independent origin. The presence of a transferable *mcr-1*-harboring IncX4 plasmid in a healthy dog with no prior colistin exposure implies the risk of silent dissemination of antimicrobial resistance.

## 1 Introduction

Antimicrobial resistance (AMR) has emerged as a critical global health challenge, threatening the efficacy of last-line antibiotics. Among these, colistin has been reintroduced as a therapeutic option against multidrug-resistant Gram-negative pathogens due to its potent bactericidal activity (1). However, the emergence of colistin-resistant bacteria, particularly those harboring mobile colistin resistance (*mcr*) genes, has raised serious concerns regarding treatment failure and the potential for widespread dissemination (2). Colistin resistance in *Escherichia coli* is mediated by both chromosomal mutations and plasmid-borne genes. Chromosome-mediated resistance is typically associated with modifications in the lipid A component of lipopolysaccharides (LPS), which reduce the negative charge and impedes colistin binding. The modification is regulated by two-component systems such as PmrAB and PhoPQ, which activate enzymes like ArnT and EptA that add 4-amino-4-deoxy-L-arabinose or phosphoethanolamine (pEtN) to lipid A, thereby conferring resistance (3). In contrast, plasmid-mediated resistance is primarily driven by the *mcr* gene family, which currently comprises *mcr*-1 to *mcr*-10. These genes encode pEtN transferases that similarly modify lipid A, but their plasmid localization facilitates horizontal gene transfer across bacterial populations and host species. Additionally, non-MCR-mediated resistance has been observed in *mcr-*deficient *E. coli* strains, suggesting alternative pathways such as chromosomal mutations in LPS biosynthesis or regulatory genes. This highlights the complexity of colistin resistance and the need for comprehensive molecular surveillance to inform treatment and containment strategies (4).

Since the first identification of *mcr-1* in *E. coli* in 2015, *mcr* genes have been reported in bacteria isolated from humans, livestock, and the environment in more than 60 countries, implicating the zoonotic potential and widespread dissemination (5, 6). Furthermore, the plasmid-mediated *mcr* genes have been detected not only in *E. coli* but also in other genera belonging to Enterobacteriaceae such as *Salmonella*, *Klebsiella*, *Kluyvera*, *Citrobacter*, and *Cronobacter*, indicating their widespread distribution across hosts (1, 7). Recent studies have demonstrated genetic similarities between *mcr*-positive isolates from humans and companion animals, suggesting possible zoonotic transmission (8). In particular, companion animals may act as reservoirs and vectors for resistant strains due to their close contact with humans and frequent exposure to antimicrobials (9). In China, resistance to colistin in *E. coli* from livestock and human sources has remarkably declined since the banning of its use as a growth promoter in 2017. However, colistin-resistant strains have been continuously detected in companion animals, along with the report of plasmid-mediated *mcr* gene transmission between pets and humans (8). These observations underscore the importance of surveillance on antimicrobial resistance in veterinary settings, particularly in countries such as South Korea, where the rapidly growing companion animal population has contributed to the increase of antimicrobial use in veterinary practice. In South Korea, the companion animal population is estimated to comprise approximately 6–7 million dogs and 2–3 million cats (10). In addition, more than 110,000 companion animals are abandoned and sent to shelters each year (11).

In this study, we characterized colistin-resistant *E. coli* isolated from a companion animal, elucidating the genetic features of plasmid-mediated *mcr*-carrying IncX4 plasmids and their potential for horizontal transmission.

## 2 Materials and methods

### 2.1 Specimen collection and isolation

A castrated male Pomeranian dog born in August 2016 presented with suspected spinal or neurological issues in April 2024. However, the dog exhibited no clinical signs of infectious disease. It was treated with a multimodal oral protocol, including gabapentin (10 mg/kg), methocarbamol (10 mg/kg), tramadol (5 mg/kg), and prednisolone (0.25 mg/kg with tapering), along with silymarin (10 mg/kg) and ursodeoxycholic acid (10 mg/kg). All prescribed medications were administered twice daily. The patient showed improvement in pain syndrome, with appetite and elimination patterns remaining stable throughout the treatment period. A follow-up examination was conducted in June 2024 to assess the patient’s condition with fecal screening. A rectal swab was collected during this visit for microbiological analysis. It was suspended in 2 mL of buffered peptone water (BD Difco, USA), and one loopful of the suspension was streaked onto MacConkey agar (BD Difco, USA). The plate was incubated at 37 °C for 18–20 h. A single red colony was isolated and subcultured for purification. The isolate was identified by polymerase chain reaction (PCR) targeting a beta-glucuronidase gene, *uidA*, and a universal stress protein marker gene, *uspA*, in *E. coli* (12). *E. coli* ATCC 25922 was used as a positive control.

### 2.2 Antimicrobial susceptibility testing

Antibiotic susceptibility of the *E. coli* strain Z1324PEC0027 was assessed using the broth microdilution method. Fresh colonies grown on MacConkey agar were suspended in 2 mL of 0.85% saline. The turbidity of the suspension was adjusted to a 0.6 McFarland standard using a densitometer (Densimat, bioMérieux, France). A 10 μL aliquot of the standardized bacterial suspension was then added to 11 mL of Sensititre Cation-Adjusted Mueller-Hinton Broth (Thermo Scientific, Remel Inc., USA) and mixed thoroughly. Fifty microliters of the resulting mixture were dispensed into each well of a Sensititre custom Gram-negative panel (KRCDC2F; TREK Diagnostic Systems Ltd., UK), sealed with a transparent film, and incubated at 36 °C for more than 18 h. The minimum inhibitory concentrations (MICs) were determined for 16 antibiotics: ampicillin (AMP), cefoxitin (FOX), cefotaxime (CTX), ceftriaxone (CRO), ceftazidime (CAZ), imipenem (IPM), streptomycin (STR), gentamicin (GEN), amikacin (AMK), nalidixic acid (NAL), ciprofloxacin (CIP), tetracycline (TET), chloramphenicol (CHL), colistin (COL), azithromycin (AZI), and trimethoprim/sulfamethoxazole (SXT). Interpretation of MIC values followed the guidelines established by the Clinical and Laboratory Standards Institute (13).

### 2.3 Detection of ESBL and colistin resistance genes

The resistance to third-generation cephalosporins of a bacterium was determined with cefotaxime and ceftriaxone. Total genomic DNA was extracted from an overnight culture using the LaboPass™ Bacteria Mini DNA purification kit (Cosmogentech, South Korea). The detection of extended-spectrum β-lactamase (ESBL) genes was performed using multiplex PCR for the presence of the *bla*_CTX-M_ gene as previously described (14). Also, PCR targeting the *mcr-1* and *mcr-2* genes was conducted to detect plasmid-mediated colistin resistance as the two variants are the most prevalent among *mcr* types identified in *E. coli* (15, 16). Amplified products were verified by agarose gel electrophoresis.

### 2.4 Conjugation assay

To evaluate the horizontal transfer of colistin resistance, conjugation experiments were performed using a broth mating method (15). Donor strain, *E. coli* Z1324PEC0027 carrying the plasmid-mediated *mcr-1* gene, was cultivated until reaching the logarithmic phase and mixed with the recipient strain, sodium azide-resistant *E. coli* J53, at a 1:1 ratio (2 mL each). The mixture was incubated at 37 °C for 18–24 h without shaking to facilitate conjugation. Following incubation, the mating mixture was serially diluted and plated onto selective media supplemented with colistin (2 μg/mL) and sodium azide (200 μg/mL) to select transconjugants. Donor or recipient strains were maintained on the media supplemented only with colistin (2 μg/mL) or sodium azide (200 μg/mL), respectively. Conjugation frequency was calculated as the ratio of colony-forming units (CFU) of transconjugants to CFU of recipient cells, expressed as the number of transconjugants per recipient cell. Plasmid transfer was confirmed by PCR amplification of the *mcr-1* gene in transconjugants.

### 2.5 Genome analysis

Whole-genome sequencing of the colistin-resistant *E. coli* strain Z1324PEC0027 was carried out by Macrogen Inc. (Seoul, South Korea) using the Revio system (Pacific Biosciences, USA). The extracted genomic DNA with high quality was used to determine long-read sequences suitable for *de novo* assembly. The raw reads were assembled with Canu (v2.2) (17), resulting in high-quality contigs. Genome annotation was performed using the Bacterial and Viral Bioinformatics Resource Center (BV-BRC, v3.28.5) (18). Antimicrobial resistance genes were identified with ResFinder (v4.1) (19). Circular genome maps were generated with ProkSee (v1.0) (20) to visualize the genomic architecture.

The plasmids identified in this study were compared with previously reported colistin-resistant plasmids (15, 16, 21–25). Publicly available sequences of IncX4 plasmids harboring *mcr-1* were retrieved from the NCBI GenBank database based on relevant literature. Accession numbers of the plasmids used for comparative analysis are listed in Supplementary Table S3. Multiple sequence alignments were performed using EasyFig (v2.2.5) (26) to assess backbone synteny and gene organization.

## 3 Results

### 3.1 Isolation and characterization of colistin-resistant ESBL-producing *E. coli*

A colistin-resistant *E. coli* strain Z1324PEC0027 was isolated from the rectal swab sample obtained from a pet dog owned by a veterinary nurse in South Korea. The dog had no previous exposure to colistin treatment, but the *E. coli* strain was isolated during a clinical examination for symptoms unrelated to infection. The unexpected detection of a colistin-resistant strain in a healthy dog suggests the potential for environmental or occupational dissemination of resistant bacteria.

The isolate was resistant to CTX (MIC = 8 μg/mL), CRO (MIC = 32 μg/mL), and colistin (MIC = 8 μg/mL). The strain was classified as multidrug-resistant (MDR) because it also exhibited resistance to multiple other antibiotics, including AMP, TET, CHL, and STR (Supplementary Table S1). The *bla*_CTX-M-55_ gene for conferring resistance to third-generation cephalosporins was detected in the ESBL-producer, which also harbored *mcr-1* responsible for colistin resistance.

### 3.2 Plasmid replicon and conjugation

**Figure 1:**
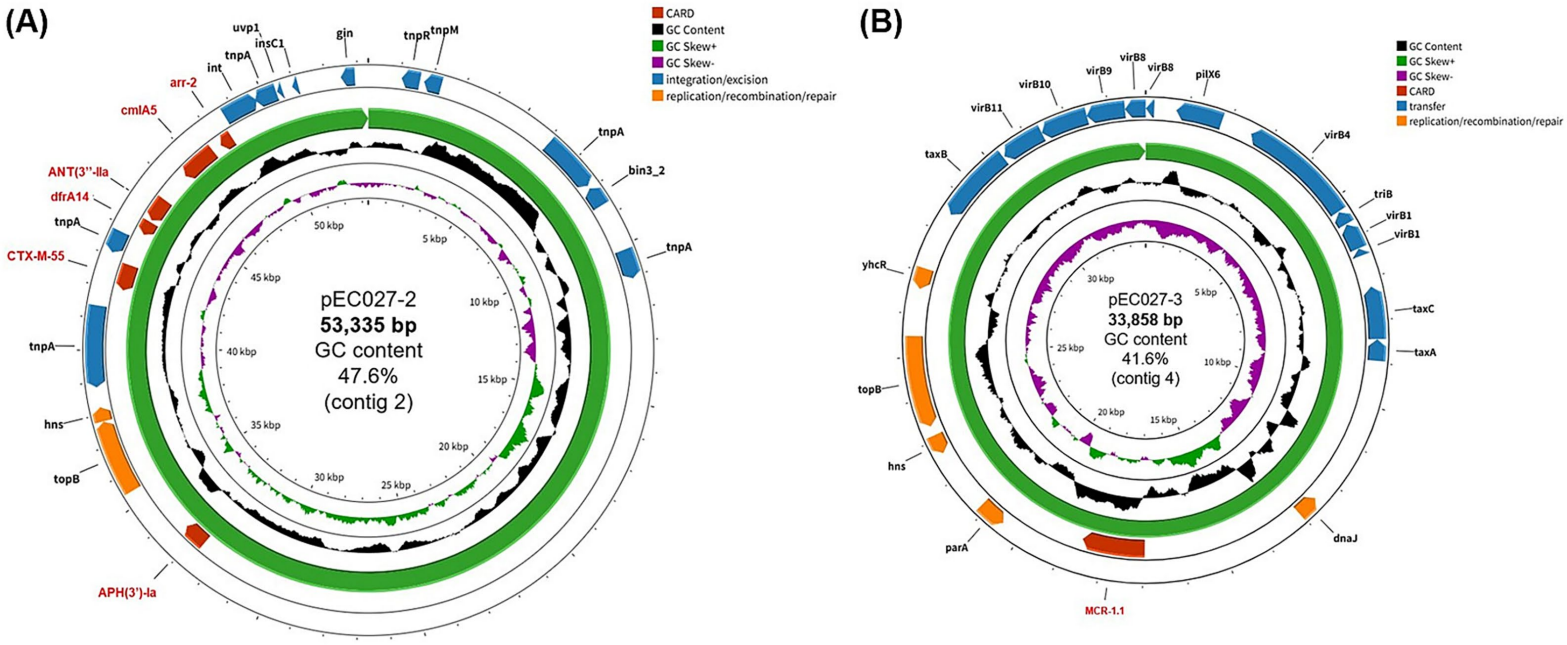
Circular map of plasmids, pEC027-2 **(A)** and pEC027-3 **(B)**, isolated from colistin-resistant *Escherichia coli* Z1324PEC0026 strain. Each map displays annotated genetic elements, including antimicrobial resistance genes (red), transfer/integration/excision-related genes (blue), and replication/recombination/repair-associated genes (orange). Concentric rings represent GC content, GC skew (positive/negative).

The genome of *E. coli* Z1324PEC0027 comprised of a chromosome and four plasmids, which was deposited in GenBank under accession numbers CP195926 to CP195930. Specifically, an IncX4-type plasmid (33,858 bp, GC content 41.6%) designated pEC027-3 (CP195929) harbored the *mcr-1* gene, while the other plasmid (53,335 bp, GC content 47.6%), pEC027-1 (CP195927), was classified as an IncX1-type and carried additional multiple resistance genes, including *bla*_CTX-M-55_*, bla*_OXA-10_*, qnrS1*, *dfrA14*, *aph(3″)-Ia*, *aadA1*, *cmlA1*, *arr-2*, and *tet(A)* (Table 1; Figures 1A,B). The remaining two plasmids, pEC027-2 (35,576 bp; GC content 48.2%) and pEC027-4 (3,793 bp; GC content 40.6%), did not contain any identifiable antimicrobial resistance genes. Conjugation assays demonstrated successful transfer of the colistin resistance phenotype to a recipient strain with a frequency of 4.64 × 10^−2^ per recipient cell, indicating the mobility of the *mcr-1*-carrying plasmid pEC027-3 under laboratory conditions (Supplementary Table S2). In contrast, other plasmids, including pEC027-1 carrying *bla*_CTX-M-55_, were not transferred to the recipient strain.

**Table 1:** Genomic features of colistin-resistant *Escherichia coli* Z1324PEC0026 describing antimicrobial resistance genes (ARGs) and plasmid replicon types.

Contig	Name (accession no.)	Size (bp)	G+C %	CDS	Inc type	Resistance gene
1	Chromosome (CP195926)	4,616,439	50.7	4,485	NA	No ARG
2	Plasmid pEC027-1 (CP195927)	53,335	47.6	66	IncX1	*bla*_CTX-M-55_, *bla*_OXA-10_, *qnrS1*, *dfrA14*, *aph(3″)-Ia*, *aadA1*, *cmlA1*, *arr-2*, *tet(A)*
3	Plasmid pEC027-2 (CP195928)	35,576	48.2	59	ND	No ARG
4	Plasmid pEC027-3 (CP195929)	33,858	41.6	54	IncX4	*mcr-1*
5	Plasmid pEC027-4 (CP195930)	3,793	40.6	7	ND	No ARG

CDS, coding DNA sequence; Inc, Incompatibility; NA, not applicable; ND, not determined.

### 3.3 Comparative genomic analysis of *mcr-1* carrying IncX4 plasmids

**Figure 2:**
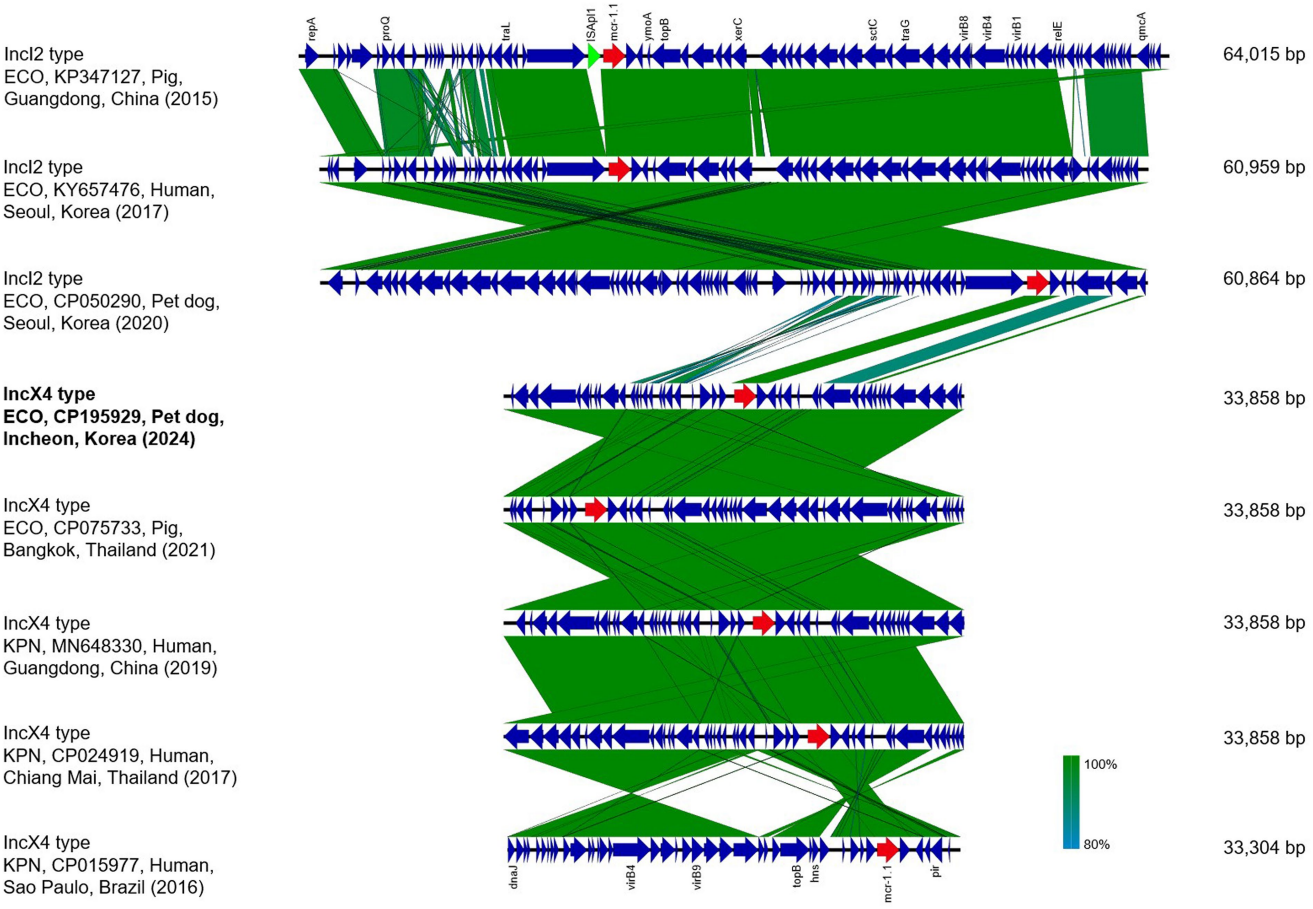
Comparative analysis of *mcr-1*-carrying plasmids in *Escherichia coli* (ECO) and *Klebsiella pneumoniae* (KPN) isolates from humans, food-producing animals, and companion animals. Regions with >95% nucleotide identity are shaded in green, indicating highly conserved backbone structures among the plasmids. Red arrows represent the colistin resistance gene (*mcr*-1), while blue arrows indicate coding sequences, including plasmid replication, mobilization, and maintenance.

The *mcr-1*-carrying IncX4 plasmid, pEC027-3, was compared with reported IncX4 plasmids to assess the genetic similarity and structural conservation (Figure 2 and Supplementary Table S3). The earliest *mcr-1*-carrying plasmid was reported in China as an IncI2 type (accession no. KP347127) in which the *mcr-1* gene was flanked by the insertion sequences, IS*Apl1*, on both sides, suggesting facilitated gene mobilization (15). In contrast, the IS*Apl1* elements were absent in the compared IncX4 plasmids, indicating that the *mcr-1* gene had been stably integrated into the plasmid backbone and its subsequent mobilization might be limited. Similarly, IS*Apl1* was not found in the plasmid pEC027-3, and the *mcr-1* gene was located without any associated mobile genetic elements. The plasmid shared a highly conserved backbone structure, exhibiting 99.8% of sequence similarity when compared with IncX4 plasmids previously identified from humans, swine, and companion animals in East and Southeast Asia (Figure 2 and Supplementary Table S3). In particular, the *mcr-1* gene was conserved with only minor variations in flanking regions among the compared plasmids. Although the IncX4 plasmids reported in Asia were found in different genera, including *E. coli* and *Klebsiella pneumoniae*, their significant genetic similarities indicated their widespread dissemination and horizontal gene transfer across microbial hosts. In contrast, a distinct genetic structure was also identified in the IncX4 plasmid from Brazil (South America). The genetic structure suggested recombination events that maintained the same gene context while leading to regional genetic divergence (Figure 2).

## 4 Discussion

The identification of a colistin-resistant *E. coli* strain in a companion dog with no prior exposure to colistin is of particular concern, as colistin is considered a last-resort antibiotic, and the presence of *mcr*-mediated resistance in household animals may facilitate the silent dissemination of AMR within veterinary clinics, households, and the broader community. The dog, owned by a veterinary nurse, presented a non-infectious spinal condition but harbored the colistin-resistant strain despite no prior antimicrobial treatment, suggesting environmental, dietary, or occupational acquisition of AMR bacteria (27). Companion animals are increasingly recognized as reservoirs and potential transmitters of multidrug-resistant microorganisms, including *mcr*-positive *E. coli*, particularly in households or veterinary environments with high antibiotic use (28, 29). The detection of colistin resistance in a domestic dog without any history of corresponding antimicrobial treatment is consistent with recent reports that resistant bacteria can circulate silently through indirect contact routes, such as exposure to shared environmental surfaces or utensils, as well as human-mediated transfer between pets and owners (27, 30).

The strain Z1324PEC0026, an ESBL-producer harboring the *mcr-1* gene on an IncX4-type plasmid, was resistant to colistin, cephalosporins, aminoglycosides, tetracyclines, and phenicols, which was consistent with the definition of multidrug resistance (MDR), acquired non-susceptibility to at least one agent in three or more antimicrobial classes (31). The worldwide spread of MDR strains poses a severe public health challenge, as infections caused by pathogens exhibiting MDR phenotypes may lead to antibiotic treatment failure. Among ESBL-producing Enterobacteriaceae, CTX-M type lactamases are currently the most prevalent and *E. coli* carrying *bla*_CTX-M-55_ has been reported globally, with a particularly high prevalence in Asia (32). The co-existence of *mcr-1* and *bla*_CTX-M-55_ in a single strain confers resistance to two last-line antibiotic classes, polymyxins and β-lactams, causing serious problems in both human and veterinary medicine. Such co-resistance has been increasingly reported in isolates from humans, animals, and environmental sources worldwide (33–35).

The IncX4-type plasmid is a representative incompatibility group that is known as a common vehicle for colistin resistance (36). IncX4 plasmids are relatively small, impose minimal fitness costs on their bacterial hosts, and have transferability at high frequencies, facilitating the spread of *mcr-1* among Enterobacteriaceae in both clinical settings and animal-associated environments, including household settings, animal shelters, farms, and veterinary clinics (37, 38). The IncX4 plasmid pEC027-3 carrying *mcr-1* in strain Z1324PEC0026 exhibited a conjugation frequency of 4.64 × 10^−2^ per recipient cell, consistent with previously reported frequencies for IncX4 plasmids ranging from 10^−2^ to 10^−4^ (39). The frequency was relatively higher than that observed in other incompatibility groups. IncX4 plasmids are generally reported to display higher conjugation frequencies than other *mcr*-carrying plasmids, such as IncI2 or IncHI2, which typically exhibit frequencies in the range of 10^−2^ to 10^−6^ per recipient cell (36, 39). Therefore, IncX4 plasmids are considered key drivers in the global dissemination of *mcr*-mediated colistin resistance, as their high transfer efficiency combined with low fitness costs enables widespread propagation among bacterial populations under favorable conditions (39, 41). The relatively high conjugation frequency observed in this study highlights the potential of these plasmids for horizontal dissemination within the gut microbiota, posing a threat to both animal and human health. The comparison of plasmid sequences showed high synteny and backbone conservation between pEC027-3 and previously reported *mcr-1*-positive IncX4 plasmids identified from humans, food-producing animals, wild mammals, and environmental sources (42–45), suggesting their broad distribution across diverse ecological niches.

Earlier studies in South Korea have reported *mcr-1*-bearing plasmids predominantly belonging to the IncI2 and IncHI2 types, identified in *E. coli* and *Enterobacter aerogenes* isolated from humans and companion animals (16, 21, 46). An *mcr-1*-positive IncI2 plasmid was found in *E. coli* obtained from a healthy dog, and its chromosomal context resembled that of human isolates in the community (21). These plasmids ranged from 60 to 250 kb in size and harbored additional resistance markers. By contrast, the IncX4 type pEC027-3 identified in this study was 33.9 kb, showing high synteny with global IncX4 *mcr-1* plasmids but possessed distinct backbone structure compared to IncI2 type plasmids found in South Korea (Figure 2). The discovery of this unique IncX4 plasmid in a companion animal with no documented antibiotic exposure suggested an increasing diversity of *mcr*-harboring plasmids in South Korea. In addition to household pets, recent studies have identified *mcr-1*–positive colistin-resistant *E. coli* in stray or free-roaming dogs, suggesting that non-household animals may also act as environmental reservoirs contributing to AMR dissemination (30, 47). Our findings demonstrated not only the capacity of highly transferable IncX4 plasmids to disseminate *mcr-1* in companion animals but also the imperative of integrated surveillance strategies to detect the silent dissemination of antimicrobial resistance across ecological boundaries.

The other plasmid pEC027-2 in strain Z1324PEC0026 carried additional antimicrobial resistance genes, *bla*_CTX-M-55_, *bla*_OXA-10_, *qnrS1*, *dfrA14*, *aph(3″)-Ia*, *aadA1*, *cmlA1*, *arr-2*, and *tet(A)*, which further contributed to the MDR phenotype. The coexistence of multiple plasmids carrying different AMR genes within a single host cell may exacerbate the spread of plasmid-mediated resistance, as co-selective pressures maintain diverse resistance traits (48).

Although the detection of *mcr-1*–carrying *E. coli* in a household pet suggests the potential for AMR dissemination at the human–animal interface, this study has limitations in conclusively demonstrating such transmission. Therefore, longitudinal cohort studies incorporating owner–pet paired surveillance are required.

## 5 Conclusion

In this study, we isolated and characterized a colistin-resistant *E. coli* from a companion dog with no history of colistin treatment in South Korea. The strain carried the plasmid-mediated *mcr-1* gene on a transferable IncX4 plasmid with high conjugation potential, indicating its capability for horizontal dissemination of colistin resistance among *E. coli* strains. The presence of *mcr*-mediated resistance in a household pet raises concerns about community-level public health, particularly at the human-animal interface where close contact occurs. These results suggest the potential for the silent dissemination of IncX4 plasmids carrying colistin resistance both clinical and non-clinical settings.

## Data Availability

The datasets presented in this study can be found in online repositories. The names of the repository/repositories and accession number(s) can be found at: https://www.ncbi.nlm.nih.gov/genbank/, CP195926, CP195927, CP195928, CP195929, and CP195930.
