# Supplementary material for: Case Report: Transferable IncX4 plasmid carrying mcr-1 in colistin-resistant Escherichia coli from a healthy pet dog in South Korea
Source: Front Vet Sci. 2026 Jan 27;12:1746399. doi: 10.3389/fvets.2025.1746399 (PMC12888783; doi:10.3389/fvets.2025.1746399)
Supplement: Supplementary file 1 [file Data_Sheet_1.pdf]

Supplemental Material

Supplemental Table S1. Antimicrobial susceptibility of colistin-resistant *E. coli* Z1324PEC0026 based on broth microdilution tests. Resistance interpretation is based on CLSI breakpoints.

Antibiotic	MIC (ug/mL)	Interpretation
Ciprofloxacin	0.25	Susceptible
Imipenem	<1	Susceptible
Colistin	8	Resistant
Cefotaxime	8	Resistant
Ampicillin	≥64	Resistant
Ceftriaxone	32	Resistant
Tetracycline	128	Resistant
Chloramphenicol	16	Resistant
Gentamicin	<4	Susceptible
Azithromycin	4	Susceptible
Streptomycin	64	Resistant
Amikacin	<4	Susceptible
Nalidixic acid	4	Susceptible
Sulfamethoxazole/Trimethoprim	<1/19	Susceptible
Cefoxitin	<4	Susceptible
Ceftazidime	2	Susceptible

Supplemental Table S2. Conjugation analysis using colistin-resistant *E. coli* Z1324PEC0026 as a donor and *E. coli* J53 as a recipient.

Antibiotic	MIC (ug/mL) of selected antibiotics		
	Donor Z1324PEC0026	Recipient J53	Transconjugant pCEC-027
Ciprofloxacin	0.25	<0.03	<0.03
Imipenem	<1	<1	<1
Colistin	8	<2	8
Cefotaxime	8	2	<1
Ampicillin	≥64	<2	4
Ceftriaxone	32	2	<1
Tetracycline	128	<2	<2
Chloramphenicol	16	8	4
Gentamicin	<4	<1	<1
Azithromycin	4	<2	<2
Streptomycin	64	8	4
Amikacin	<4	<4	<4
Nalidixic acid	4	<2	4
Sulfamethoxazole/ Trimethoprim	<1/19	<1/19	<1/19
Cefoxitin	<4	<4	<4
Ceftazidime	2	4	1
CFU/mL	1.5×10^{6}	2.8×10^{5}	1.3×10^{4}
Conjugation frequency [a]	4.64×10^{-2}		

[a] Calculated as (CFU of transconjugant / CFU recipient) × 100.

Supplemental Table S3. Compared plasmids carrying the *mcr-1* gene.

Bacteria (origin)	Plasmid	Replicon type	Size (bp)	Accession number	Country	Reference
Escherichia coli (Pig)	pHNSHP45	IncI2	64,015	KP347127	China	[15]
Escherichia coli (Human)	pCEC-527_4	IncI2	60,959	KY657476	South Korea	[16]
Escherichia coli (Dog)	pK19EC149	IncI2	60,864	CP050290	South Korea	[43]
Escherichia coli (Dog)	pEC027-3	IncX4	33,858	CP195929	South Korea	This study
Escherichia coli (Pig)	pCP52E	IncX4	33,858	CP075733	Thailand	[47]
Klebsiella pneumoniae (Human)	pT38	IncX4	33,858	MN648330	China	[48]
Klebsiella pneumoniae (Human)	pKPNH54.3	IncX4	33,858	CP024919	Thailand	[49]
Escherichia coli (Human)	plCBEC72Hmcr	IncX4	33,304	CP015977	Brazil	[50]
